# Discrete cortical representations and their stability in the presence of synaptic turnover

**DOI:** 10.1186/1471-2202-16-S1-P114

**Published:** 2015-12-18

**Authors:** Bastian Eppler, Dominik Aschauer, Simon Rumpel, Matthias Kaschube

**Affiliations:** 1 Frankfurt Institute for Advanced Studies, 60438 Frankfurt, Germany; 2 Goethe-Universität, 60438 Frankfurt, Germany; 3 Johannes-Gutenberg-Universität, 55122 Mainz, Germany

## 

Population imaging in mouse auditory cortex revealed clustering of neural responses to brief complex sounds: the activity of a local population typically falls close to one out of a small number of observed states [1]. These clusters appear to group sets of auditory stimuli into a discrete set of activity patterns and could thereby form the basis for representations of sound categories. However, to be useful for the brain, such representations should be robust against fluctuations in the underlying circuitry, which are significant even in the absences of any explicit learning paradigm [2]. Here we introduce a novel firing rate based circuit model of mouse auditory cortex to study the emergence of the observed activity cluster states and their structural stability in the presence of synaptic noise. We find that generic random networks by virtue of their inhibitory recurrent connectivity can group complex sounds spontaneously into essentially discrete sets of activity states. Moreover, these states can display high degrees of stability, even when modifying a substantial fraction of synaptic connections, as long as the basic statistics of connectivity is maintained. We use the insights gained from the analysis of our model to interpret data gathered in a parallel effort, employing chronic two-photon imaging of population activity in the auditory cortex of awake mice.
